# Development of an activity disease score in patients with uveitis (UVEDAI)

**DOI:** 10.1007/s00296-016-3593-1

**Published:** 2016-11-04

**Authors:** Esperanza Pato, Mª Auxiliadora Martin-Martinez, Adela Castelló, Rosalía Méndez-Fernandez, Santiago Muñoz-Fernández, Miguel Cordero-Coma, Lucia Martinez-Costa, Elia Valls, Miguel Reyes, Félix Francisco, Mar Esteban, Alex Fonollosa, Fernando Sanchez-Alonso, Cruz Fernández-Espartero, Teresa Diaz-Valle, José Miguel Carrasco, Emma Beltran-Catalán, Marisa Hernández-Garfella, María Victoria Hernández, Laura Pelegrin, Ricardo Blanco, David Diaz-Valle

**Affiliations:** 1 Rheumatology Department and Health Research Institute (IdISSC), Hospital Clínico San Carlos, C/ Profesor Martin Lagos s/n, 28040 Madrid, Spain; 2 Research Unit of Spanish Society of Rheumatology, Madrid, Spain; 3 Cancer Epidemiology Unit, National Center for Epidemiology, Instituto de Salud Carlos III, Madrid, Spain; 4 Ophthalmology Department and Health Research Institute (IdISSC), Hospital Clínico San Carlos, Madrid, Spain; 5 Cooperative Research Network on Age-Related Ocular Pathology, Visual and Life Quality, Instituto de Salud Carlos III, Madrid, Spain; 6 Rheumatology Department, Hospital Universitario Infanta Sofia, Universidad Europea de Madrid, San Sebastian de los Reyes, Madrid, Spain; 7 Ophthalmolgy Department, Hospital Universitario de León, León, Spain; 8 Ophthalmolgy Department, Hospital Universitario Doctor Peset, Valencia, Spain; 9 Rheumatology Department, Hospital Universitario Doctor Peset, Valencia, Spain; 10 Ophthalmolgy Department, Hospital de Gran Canaria Doctor Negrin, Gran Canaria, Spain; 11 Rheumatology Department, Hospital de Gran Canaria Doctor Negrin, Gran Canaria, Spain; 12 Ophthalmology Department, Hospital Universitario Infanta Sofia, San Sebastian de los Reyes, Madrid, Spain; 13 Ophthalmology Department, BioCruces Health Research Institute, Cruces University Hospital, University of the Basque Country, Barakaldo, Spain; 14 Rheumatoloy Department, Hospital Universitario de Móstoles, Móstoles, Madrid, Spain; 15 Ophthalmology Department, Hospital Universitario de Móstoles, Móstoles, Madrid, Spain; 16 ATLANTES Research Programme, Institute for Culture and Society, University of Navarra, Pamplona, Spain; 17 Rheumatoloy Department, Hospital General Universitario de Valencia, Valencia, Spain; 18 Ophthalmology Department, Hospital General Universitario de Valencia, Valencia, Spain; 19 Rheumatology Department, Hospital Clínic of Barcelona, Barcelona, Spain; 20 Ophthalmology Department, Hospital Clínic of Barcelona, Barcelona, Spain; 21 Rheumatology Department, Hospital Universitario de Valdecilla, Santander, Spain

**Keywords:** Uveitis, Activity index, Ocular inflammation, Outcome measures

## Abstract

To develop a disease activity index for patients with uveitis (UVEDAI) encompassing the relevant domains of disease activity considered important among experts in this field. The steps for designing UVEDAI were: (a) Defining the construct and establishing the domains through a formal judgment of experts, (b) A two-round Delphi study with a panel of 15 experts to determine the relevant items, (c) Selection of items: A logistic regression model was developed that set ocular inflammatory activity as the dependent variable. The construct “uveitis inflammatory activity” was defined as any intraocular inflammation that included external structures (cornea) in addition to uvea. Seven domains and 15 items were identified: best-corrected visual acuity, inflammation of the anterior chamber (anterior chamber cells, hypopyon, the presence of fibrin, active posterior keratic precipitates and iris nodules), intraocular pressure, inflammation of the vitreous cavity (vitreous haze, snowballs and snowbanks), central macular edema, inflammation of the posterior pole (the presence and number of choroidal/retinal lesions, vascular inflammation and papillitis), and global assessment from both (patient and physician). From all the variables studied in the multivariate model, anterior chamber cell grade, vitreous haze, central macular edema, inflammatory vessel sheathing, papillitis, choroidal/retinal lesions and patient evaluation were included in UVEDAI. UVEDAI is an index designed to assess the global ocular inflammatory activity in patients with uveitis. It might prove worthwhile to motorize the activity of this extraarticular manifestation of some rheumatic diseases.

**Electronic supplementary material:**

The online version of this article (doi:10.1007/s00296-016-3593-1) contains supplementary material, which is available to authorized users.

## Introduction

Uveitis is defined as inflammation of the uvea, the vascular middle layer of the eye. The term refers to a great number of diseases characterized by intraocular inflammation involving the uvea and other ocular structures (retina, vitreous cavity and retinal vessels) [1]. It may be characterized as having a poor visual prognosis and remains one of the leading blinding disorders [2, 3]. Uveitis is an extraarticular manifestation frequently found in many rheumatic diseases (spondyloarthropathy, Behçet, sarcoidosis, lupus, vasculitis, etc.) and may cause significant morbidity. In some patients, such as those with Behçet disease or spondyloarthritis, uveitis is often the origin of the main symptoms of the disease. Consequently, the implementation of interdisciplinary units—ophthalmology and rheumatology—assumes considerable relevance in the assessment and management of these patients.

The Standardization of Uveitis Nomenclature Working Group [4, 5] (SUN) developed criteria based on the primary anatomic location of inflammation within the eye (International Uveitis Study Group [6]), as well as criteria for onset, duration, course and disease activity. The SUN criteria for the classification of uveitis have become the standard when publishing uveitis data, ensuring greater analytical rigor when comparing studies. However, such criteria only take into account inflammation in the anterior chamber and vitreous haze when defining uveitis activity.

Standardized and validated outcome measures of disease activity are lacking in uveitis management, which makes it difficult to compare efficacy and response to treatment [7–9]. In addition, the activity of uveitis is not recorded by the usual articular indexes that are used by rheumatologists to monitoring disease activity and function such as BASDAI, BASFI or ASDAS. In this line, Denniston et al. [9] carried out a systematic review of clinical trial or treatments for uveitis, highlighting the heterogeneity of primary outcomes and arguing that the complex issue of selecting outcome measures for clinical trials of efficacy related to uveitis needs to be addressed.

As in rheumatology, the validation of outcome measures and the development of a disease activity index [10–12] can be a major advance in clinical trials as well as in evaluation of treatment response of patients with uveitis in daily clinical practice.

The objectives of the current study were to develop a disease activity index for patients with uveitis (UVEDAI) that would include relevant domains of disease activity considered important among experts in uveitis.

## Methods

### Development of UVEDAI

The index has been developed according to the criteria described in the Outcome Measures in Rheumatology Clinical Trials (OMERACT) [13].

A formal interdisciplinary working group for uveitis, consisting of eight ophthalmologists and seven rheumatologists, was formed. The participants had relevant experience in the management of all types of uveitis and work in interdisciplinary uveitis units at hospitals in the Spanish national health system. Chronological steps to design UVEDAI:

1. Definition of the construct and establishment of domains: The consensus technique used was the formal judgment of experts, which represents the informed opinion of people with experience in the subject, who are recognized by others as skilled in the topic, and who can provide information, evidence, judgments and assessments [14]. A consensus meeting took place in October 2011 in Madrid, coordinated by a methodologist with training and experienced in this technique, and all the members participated. First, one or two indices of activity, based on the anatomic location, were discussed, which the expert panel decided by consensus to build into a global index. Second, the construct “uveitis inflammatory activity” was defined as any intraocular inflammation including external structures (cornea) in addition to uvea, and the domains and items were established. Seven domains were identified: best-corrected visual acuity (BCVA), inflammation of the anterior chamber, intraocular pressure, inflammation of the vitreous cavity, central macular edema (ME), inflammation of the posterior pole and global assessment.
2. Determination of items: The panel selected 15 relevant items that would thereafter be tested to derive the new disease activity index. A two-round Delphi consensus technique was used by the panel to achieve consensus. Agreement on the utility of each item to assess “uveitis inflammatory activity” was evaluated using a Likert scale (1 [strongly disagree] to 5 [strongly agree]). The degree of agreement (DA) among the working group for each item was high (DA > 85%). In the first round, 12 items reached DA > 85% and the three remaining reached DA > 85% after the second round, and thus were included in UVEDAI.
  As result, seven domains and 15 items were identified: best-corrected visual acuity, inflammation of the anterior chamber (anterior chamber (AC) cells, hypopyon, the presence of fibrin, active posterior keratic precipitates and iris nodules: five items), intraocular pressure, inflammation of the vitreous cavity (vitreous haze, snowballs and snowbanks: three items), central macular edema, inflammation of the posterior pole (the presence and number of choroidal/retinal lesions, vascular inflammation and papillitis: three items) and global assessment from both the patient and physician (two items).
3. Selection of items: A logistic regression model was constructed, the ordinal dependent variable of which was ocular inflammatory activity (with three categories: mild, moderate and severe) and as variables predictive, the items mentioned above. After building the first model, the variables were eliminated in inverse order of their significance level to obtain the minimal model explaining the maximum variability. The capability of the final model to discriminate patients with uveitis according to the level of inflammatory activity was then determined.

### Items and operational definitions

Inflammatory activity of uveitis was considered the primary endpoint. This variable was defined as any intraocular inflammation, and it was categorized as mild, moderate and severe at the discretion of the ophthalmologist following patient assessment. The manner in which each hospital carried out the evaluations was homogenized, and the CIRRUS spectral domain (Carl Zeiss) was the optical coherence tomography (SD-OCT) equipment used in all centers.

Clinical evaluations included visual acuity (best-corrected Snellen visual acuity) and ophthalmic examination. A slit-lamp examination was used to evaluate the anterior segments. AC cells were graded according to the classification established by the SUN [4]. Intraocular pressure [15] was determined by using a contact tonometer (Goldmann). Indirect ophthalmoscopy was also performed in all patients to evaluate the vitreous and posterior segments [16]. The Nussenblatt scale [17] was adapted for any vitreous haze that had been graded as mild or severe (1–2+ or 3–4+, respectively). SD-OCT was used in all patients to determine the presence of macular edema [18], which was defined as a central retinal thickness greater than 315 μm. The 1-mm central retinal thickness was evaluated using the macular cube strategy.

An overall assessment of the disease by both patient and researcher was made using a visual analog scale (VAS) of 0–10 cm, 0 being the lowest (best) possible disease activity and 10 the highest (worst). VAS assessments of patient and physician were performed with the following questions; VAS patient: “at the present time, how would you rate your eyesight using both eyes (with glasses or contact lenses, if you wear them): excellent, good, fair, poor, or very poor, or are you completely blind”. VAS physician: “taking into account eye inflammation and macular edema, indicate your assessment of disease activity experienced by this patient”.

### Selection and recruitment of patients

Patients diagnosed with uveitis at nine multidisciplinary uveitis units in hospitals from Spanish National Health System were invited to participate in the study. The recruitment period was from March 2013 to July 2014. Consecutive patients were eligible for inclusion during the recruitment period, with one out of two patients who met the selection criteria being enrolled until the sample size was reached. Inclusion criteria consisted of the following: signed informed consent by the patient or his/her legal representative, aged five years or older, patients diagnosed with active anterior, intermediate, posterior uveitis or panuveitis in at least one eye, and with active uveitis in at least one eye at the time of the selection visit. We excluded patients in complete uveitis remission, those who had been diagnosed with surgical or traumatic endophthalmitis, and any patients then participating in a clinical trial or research project related to this or other health problems. For those patients with bilateral uveitis, we took into account the fact that treatment decisions would always be based on the most inflamed eye. This study was performed following the principles outlined in the Helsinki Declaration, and the study protocol was approved by the ethics committee at all participating hospitals.

The sample size was calculated based on at least 10 events per variable, as suggested in the current literature [19–21]. The presence of moderate or severe uveitis was regarded as an event. When the expected prevalence of such an event reached 60%, we determined that 200 patients would be sufficient for designing a model that would include up to 12 variables (200 patients × 60% = 120 expected events, which corresponds to 120/10 possible variables).

### Statistical analysis

The categorical variables were described using frequencies and percentages. These were compared among activity levels using Chi-squared tests, and Fisher’s exact tests otherwise. The variables that followed a normal distribution were described using the mean and standard deviation (SD), and the differences between groups of activity were assessed using ANOVA tests. Non-normally distributed variables were described with the median and interquartile interval (IQR), and differences between groups were assessed with the Kruskal–Wallis nonparametric test. In all the analyses, a *p* value <0.05 was considered statistically significant.

Given the ordinal nature of the outcome variable (uveitis: mild, moderate and severe), we adjusted the model using ordinal logistic regression. We followed the stepwise methodology, including the potentially explanatory variables in the model, by statistical significance order. The selection of independent variables in the multivariate models was based on clinical judgments and those with a *p* value <0.20 in the bivariate analysis. After building the first model, we tested the deletion of each variable in inverse significance order to obtain the minimum model that would explain the maximum variability. The inclusion or exclusion of each variable was determined using a likelihood ratio test (LRT) with a 90% confidence level. The assumption of proportional odds across responses underlying the ordered logistic models was also verified using the approximate LRT with the same confidence level. Confidence intervals for the odds ratio were calculated at a 95% confidence level.

Regression coefficients in logarithmic format for the multivariate model were used to calculate the score used to define the ocular activity level of a given patient:

$${\text{UVEDAI}} = \mathop \sum \limits_{i = 1}^{v} \beta_{i} \cdot x_{i} \;\begin{array}{c} v = {\text{Number\,of\,variables\,included}}\\ \beta_{i} = {\text{Logarithmic\,regression\,coefficient\,for\,variable}}\,i\\ x_{i} = {\text{Variable}}\, i \\ \end{array}$$

We used the resulting score to calculate a patient’s probability of being classified with mild, moderate or severe uveitis based on the following cumulative probabilities:

$$\begin{array}{*{20}l} {Pr\left( {{\text{uveitis}} < {\text{moderate}}} \right) = \left[ {1/\left( {1~ + ~e^{{({\text{score}} - {\text{constant}}\,{\text{ mild }}\,{\text{to }}\,{\text{moderate}})}} } \right)} \right]~ = ~1/\left( {1~ + ~e^{{({\text{score}} - 1.01)}} } \right)} \hfill \\ {Pr\left( {{\text{uveitis}} < {\text{severe}}} \right) = \left[ {1/\left( {1~ + ~e^{{({\text{score}} - {\text{constant}}\,{\text{ moderate }}\,{\text{to}}\,{\text{ severe}})}} } \right)} \right]~ = ~1/\left( {1~ + ~e^{{({\text{score}} - 4.91)}} } \right)} \hfill \\ \end{array}$$

From which we calculated probabilities for the three categories:

$$\begin{aligned} &Pr({\text{uveitis}} = {\text{Mild}}) = Pr({\text{uveitis}} <{\text{Moderate}}) \\ &{Pr}({\text{uveitis}} = {\text{Moderate}}) = Pr({\text{uveitis}}<{\text{Severe}}) - Pr({\text{uveitis}} <{\text{Moderate}}) \\ &{Pr}({\text{uveitis}} = {\text{Severe}}) = 1 - Pr({\text{uveitis}} <{\text{Severe}})\end{aligned}$$

The patient was then classified in that category with the higher probability (see example in Supplementary Material).

The modeling accuracy was assessed using the area under the curve (AUC) [22–24]. To overcome any limitations in the use of AUC for binary outcomes, and basing our calculations on proportional odds, we formulated two AUC measures of uveitis: one for discriminating mild versus moderate and severe activity levels and another for mild and moderate versus severe activity. In order to provide a measurement of the uncertainty of the model’s accuracy, we calculated the 95% percentile confidence intervals for the estimation of both AUCs by performing a nonparametric bootstrap estimation with 1000 replications. Using sampling replacement, the bootstrap obtained 1000 replicates of the original dataset and coefficients of the final model. The corresponding AUCs were then estimated over each of these replicates. The 95% percentile confidence intervals for each AUC were represented by percentiles 2.5 and 97.5 of the 1000 bootstrap point estimates’ distribution.

Additionally, the internal validity of the results was assessed by comparing the estimation of the AUC with an empirical distribution of the outcome in order to determine the probability of obtaining a better model accuracy by chance. For that purpose, we calculated 1000 replicates of the original outcome variable containing the exact number of cases of mild (*n* = 77), moderate (*n* = 93) and severe (*n* = 25) uveitis, but randomly distributed. The discrimination of mild versus moderate + severe diagnosis and the discrimination of mild + moderate versus severe diagnosis were assessed for each of the 1000 random replicates by calculating the AUC. The probability of obtaining by chance an AUC better than the original was calculated, dividing by 1000 the total number of cases in which the random AUC was better than the original AUC.

## Results

Our initial sample contained 203 patients. After excluding missing values (*n* = 8), a total of 195 patients were included in the study. Demographic characteristics and bivariate analysis are summarized in Table 1. The mean age was 45.8±16.1 years; 54% (*n* = 106) were female. Forty-eight percent (*n* = 93) presented moderate activity, 39% (*n* = 77) mild activity, and 13% (*n* = 25) severe activity.

**Table 1:** Description of demographic characteristics and bivariate analysis

Variables	Activity levels				
	All (*N* = 195)	Mild (*N* = 77)	Moderate (*N* = 93)	Severe (*N* = 25)	*p*
Sex, female, *n* (%)	106 (54)	42 (55)	52 (56)	12 (48)	0.770
Age, mean (SD^a^)	45.8 (16.1)	46.5 (15.7)	45.3 (17.1)	45.4 (13.8)	0.890
Patient VAS^b^ median (IQR^c^)	5.0 (2.0–7.0)	3.0 (2.0–6.0)	5.0 (2.0–7.0)	7.0 (6.0–8.0)	<0.001
Physician VAS^b^ median (IQR^c^)	4.0 (2.0–6.0)	2.0 (2.0–3.0)	5.0 (3.0–6.0)	7.0 (6.0–8.0)	<0.001
Eye, right *n* (%)	106 (54)	40 (52)	54 (58)	12 (48)	0.570
Anatomic location *n* (%)					
Anterior	100 (51.28)	56 (72.73)	37 (39.78)	7 (28)	<0.001
Intermediate	30 (15.38)	7 (9.09)	23 (24.73)	0 (0)	
Posterior	29 (14.87)	3 (3.9)	17 (18.28)	9 (36)	
Panuveitis	36 (18.46)	11 (14.29)	16 (17.2)	9 (36)	
Visual acuity median (IQR^c^)	0.8 (0.4–1.2)	1.0 (0.5–1.2)	0.6 (0.3–1.0)	0.4 (0.2–0.8)	<0.001
Anterior chamber cell grade *n* (%)					<0.001
0	49 (25)	9 (12)	32 (34)	8 (32)	
1+	59 (30)	36 (47)	18 (19)	5 (20)	
2+	48 (25)	26 (34)	21 (23)	1 (4)	
3+	32 (16)	6 (8)	20 (22)	6 (24)	
4+	7 (4)	0 (0)	2 (2)	5 (20)	
Hypopyon median (IQR^c^)	0.0 (0.0–0.0)	0.0 (0.0–0.0)	0.0 (0.0–0.0)	0.0 (0.0–0.0)	0.620
Fibrin in the anterior chamber *n* (%)	18 (9)	4 (5)	10 (11)	4 (16)	0.197
Keratic precipitates *n* (%)	104 (53)	42 (55)	48 (52)	14 (56)	0.890
Iris nodules *n* (%)	6 (3)	4 (5)	2 (2)	0 (0)	0.500
Intraocular pressure *n* (%)					0.013
≤21 mmHg	178 (91)	73 (95)	86 (92)	19 (76)	
>21 mmHg	17 (9)	4 (5)	7 (8)	6 (24)	
Vitreous haze *n* (%)					<0.001
Null	117 (60)	59 (77)	49 (53)	9 (36)	
Mild (1–2+)	49 (25)	15 (19)	27 (29)	7 (28)	
Severe (3–4+)	26 (13)	3 (4)	16 (17)	7 (28)	
Presence of snowballs *n* (%)	23 (12)	6 (8)	15 (16)	2 (8)	0.220
Snowbanks: number of clock hours, median (IQR^c^)	0.0 (0.0–0.0)	0.0 (0.0–0.0)	0.0 (0.0–0.0)	0.0 (0.0–0.0)	0.640
Macular edema *n* (%)					<0.001
≤315 μm	155 (79)	72 (94)	63 (68)	20 (80)	
>315 μm	40 (21)	5 (6)	30 (32)	5 (20)	
Choroidal or retinal lesions: maximal size (disk diameter) median (IQR^c^)	0.0 (0.0–0.0)	0.0 (0.0–0.0)	0.0 (0.0–0.0)	0.0 (0.0–0.3)	0.198
Number of choroidal or retinal lesions *n* (%)					0.009
0	166 (85)	72 (94)	77 (83)	17 (68)	
1–5	16 (8)	4 (5)	9 (10)	3 (12)	
≥6	12 (6)	1 (1)	7 (8)	4 (16)	
Exudative retinal detachment *n* (%)	4 (2)	0 (0)	4 (4)	0 (0)	0.185
Inflammatory vessel sheathing *n* (%)	38 (19)	7 (9.1)	19 (20.4)	12 (48)	<0.001
Papillitis *n* (%)	18 (9)	2 (2.6)	10 (10.8)	6 (24)	0.004

^a^ *SD* standard deviation, ^b^ *VAS* visual analog scale, ^c^ *IQR* interquartile interval

The results of the regression model summarized in Table 2 revealed that the uveitis activity levels were more severe in patients with high degrees of anterior chamber cell grade, high degrees of vitreous haze, central macular edema over 315μm, who presented inflammatory vessel sheathing, papillitis, an elevated number of choroidal/retinal lesions and higher patient evaluation.

$$\begin{aligned} {\text{UVEDAI}} & = \left\{ {\begin{array}{*{20}c} {\begin{array}{*{20}c} { 0.00} & {{\text{if}}\,{\text{ACC}} = 0} \\ { - 1.41} & {{\text{if}}\,{\text{ACC}} = 1} \\ { - 0.72} & {{\text{if}}\,{\text{ACC}} = 2} \\ \end{array} } \\ {\begin{array}{*{20}c} { 1.09} & {{\text{if}}\,{\text{ACC}} = 3} \\ { 3.33} & {{\text{if}}\,{\text{ACC}} = 4} \\ \end{array} } \\ \end{array} } \right\} + \left\{ {\begin{array}{*{20}c} {0.00} & {{\text{if}}\,{\text{VH}} = {\text{null}}} \\ {0.38} & {{\text{if}}\,{\text{VH}} = {\text{mild}}} \\ {1.37 } & {{\text{if}}\,{\text{VH}} = {\text{severe}}} \\ \end{array} } \right\} + \left\{ {\begin{array}{*{20}c} {0.00} & {{\text{if}}\,{\text{ME}} \le 315} \\ {1.28} & {{\text{if}}\,{\text{ME}} > 315} \\ \end{array} } \right\} \\ & \quad + \left\{ {\begin{array}{*{20}c} {0.00} & {{\text{if}}\,{\text{VS}} = {\text{no}}} \\ {1.49} & {{\text{if}}\,{\text{VS}} = {\text{yes}}} \\ \end{array} } \right\} + 0.21x{\text{PE}} + \left\{ {\begin{array}{*{20}c} {0.00} & {{\text{if}}\,{\text{papilitis}} = {\text{no}}} \\ {1.40} & {{\text{if}}\,{\text{papilitis}} = {\text{yes}}} \\ \end{array} } \right\} \\ & \quad + \left\{ {\begin{array}{*{20}c} {0.00} & {{\text{if}}\,{\text{no}}.\,{\text{RL}} = 0} \\ {0.69} & {{\text{if}}\,{\text{no}}. \,{\text{RL}} = 1\, {\text{to}}\, 5} \\ { 1.61 } & {{\text{if}}\,{\text{no}}. \,{\text{RL}} \ge 6} \\ \end{array} } \right\} \\ \end{aligned}$$

ACC: anterior chamber cell grade; VH: vitreous haze; VS: inflammatory vessel sheathing; PE: patient evaluation; RE: choroidal/retinal lesions.

**Table 2:** Multivariate model of uveitis activity level in the study population

Variables	OR_adjusted_ (95% CI)	LOG (OR^a^)
Anterior chamber cell grade (ref. 0)		
1+	0.24 (0.10–0.60)	−1.41
2+	0.49 (0.19–1.27)	−0.72
3+	2.97 (1.04–8.49)	1.09
4+	27.85 (3.42–226.75)	3.33
Vitreous haze (ref. Null)		
Mild (1–2+)	1.46 (0.65–3.27)	0.38
Severe (3–4+)	3.95 (1.40–11.16)	1.37
Macular edema, μm (ref. ≤315 μm)	3.58 (1.56–8.21)	1.28
Inflammatory vessel sheathing (ref. No)	4.43 (1.71–11.53)	1.49
Patient evaluation	1.23 (1.09–1.39)	0.21
Papillitis (ref. No)	4.05 (1.18–13.92)	1.40
Number of choroidal or retinal lesions (ref. 0)		
1–5	2.00 (0.63–6.35)	0.69
≥6	4.99 (1.08–23.01)	1.61
Constant		
Mild to moderate		1.01
Moderate to severe		4.91

^a^ *LOG (OR)* logistic regression (odds ratio)

**Fig. 1:**
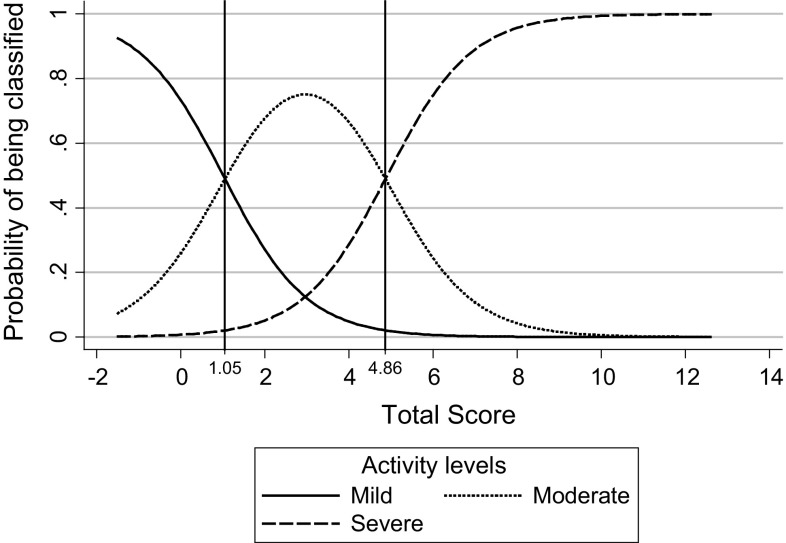
Probability of being classified with mild, moderate or severe uveitis according to the total score obtained

The distribution of the probabilities of being classified in each of the 3 uveitis categories—based on the score obtained and the cut points for each category—is shown in Fig. 1. We plotted all of the possible values for the total score along the X-axis and the probability associated with each score along the Y-axis. The three curves represent the fluctuation in probability and the cross-point between curves that allowed for the determination of a score threshold from which a uveitis status could be assigned (see Supplementary Material for an example of calculations). The range of possible values for the scores was approximately −1 × 41 to 12 × 580. As can be observed in the graph, patients with a total score below 1 × 05 will be classified in the category of mild uveitis; those with a total score between 1·06 and 4·86 will be classified as having moderate uveitis; and those with a total score greater or equal to 4 × 87 will be classified as having severe uveitis.

Therefore, clinicians using this tool will only need to substitute the values for AC cell grade, vitreous haze, macular edema, inflammatory vessel sheathing, patient evaluation, papillitis and the number of choroidal/retinal lesions in the formula given, and with the score obtained, to classify a patient’s uveitis disease activity level according to the established cut points: ≤1 × 05: mild; 1 × 06 to ≤4 × 86: moderate; ≥4 × 87: severe.

**Fig. 2:**
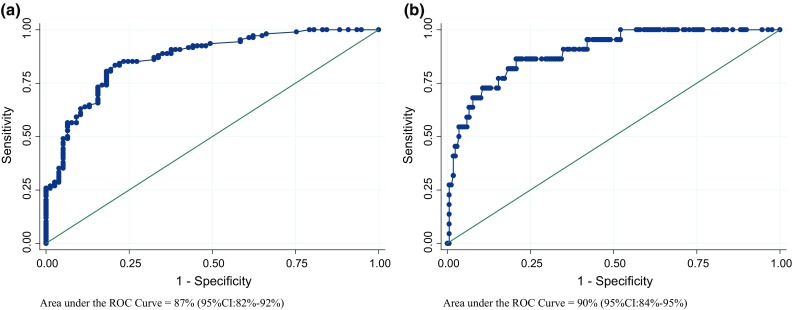
Receiver operating characteristic (ROC) curve and AUC for the predictive modeling accuracy to discriminate. **a** Patients with mild UVEITIS from patients with moderate or severe UVEITIS, **b** patients with mild or moderate UVEITIS from patients with severe UVEITIS

The discriminatory capacity of the resulting multivariate model was 87% (95% CI 82–92%) for differentiating patients with mild uveitis from those with moderate or severe uveitis and 90% (95% CI 84–95%) for differentiating patients with mild or moderate uveitis from those with severe uveitis (Fig. 2). Additionally the results from the analysis for the internal validation revealed that for none of the 1000 replicates of the random distribution of the outcome the resulting AUC was better than the original, meaning the probability of obtaining by chance an AUC better than the obtained with the original model is <0.001.

## Discussion

In our knowledge, this is the first study in which it is developed an activity index (UVEDAI) that attempts to classify patients with uveitis according to the degree of their ocular inflammatory activity. The results reported herein could be of interest to ophthalmologists, rheumatologists and other medical specialties that keep abreast of clinical investigation into systemic diseases with ocular involvement. Of all the variables investigated, anterior chamber cell grade, vitreous haze, central macular edema, inflammatory vessel sheathing, papillitis, choroidal/retinal lesions and patient evaluation were the most important to determining the activity of uveitis. Our score showed an 87% capacity to differentiate patients with mild uveitis from those with moderate or severe uveitis and an 88% capacity for differentiating patients with mild or moderate uveitis from those with severe uveitis.

Uveitis occurs in many patients who have a systemic rheumatic disease. In those with an established diagnosis of autoimmune disease, ocular inflammation can mark the severity of the systemic condition. We believe that assessment of ocular activity as an index can be useful in the global management of patients with systemic inflammatory diseases, especially in the assessment of treatment response.

The evaluation of cells in the anterior chamber is the accepted standard procedure for assessing inflammatory activity in the anterior segment [4, 25]. It is worth remarking the protective effect of categories 1 (OR_adjusted_ 0.24; 95% CI 0.10–0.60) and 2 (OR_adjusted_ 0.49; 95% CI 0.19–1.27) of this variable in the multivariate model. The explanation of this effect is that 91.84% of the patients with grade 0 have intermediate uveitis, posterior or panuveitis not anterior uveitis. In these patients, the overall value of the UVEDAI is not affected because it is corrected from the coefficients of other variables that are involved, such as macular edema, vasculitis or others. Vitreous haze is the most widely accepted measure of inflammation in the vitreous cavity. This variable has often been used as a primary outcome in many randomized clinical trials [26–29]. Other significant factor on said score was macular edema, the most common cause of irreversible visual impairment in patients with uveitis but a treatable entity [30–33]. We believe that its clinical relevance, its relationship with chronic inflammatory activity and the ease of its implementation in an objective and reproducible way with SD-OCT confirm the importance of its inclusion in UVEDAI. In the posterior pole, the presence of inflammatory vessel sheathing, choroidal/retinal lesions and papillitis has been established and characterized clinical variables indicating inflammatory activity and their presence is often a warning sign. Although in our study the number of patients with these inflammatory events was reduced, they were nonetheless significant. According to this finding, and in line with the new clinical trials, which has incorporated the above-mentioned parameters for the routine evaluation of clinical activity in uveitis [29], we regard these variables as truly significant in the composite index that has been developed. Another item bearing an important association is the patient evaluation.

On the other hand, variables, such as visual acuity, intraocular pressure, hypopyon, which are clinically relevant in the routine assessment of patient with uveitis, have not proven to be statistically significant in our study. In the same way, however, they are clinical signs that must be routinely assessed in the evaluation of any eye with uveitis.

The inclusion of the SD-OCT in the assessment of macular edema derives from its wide availability in ophthalmologist outpatient clinics, its noninvasive approach, and the fact that it is considered the gold standard in diagnosing and monitoring of ME [31, 34].

The main strength of the developed UVEDAI is its usefulness as a standardized tool that relies on variables extracted from examinations established in routine clinical practice, without complex equipment-based diagnoses. We believe that this composite index, which assesses global ocular inflammatory activity and classification, will not only allow clinicians to compare the uveitis activity, regardless of cause, but will also serve as a useful tool for future studies and trials when sensitivity to change will be analyzed. Just as in other disease activity score [11], UVEDAI assigns different weightings to each variable based on any of the ocular anatomic regions. Since these are often affected simultaneously, the result is a score that indicates global ocular inflammatory activity, which can accurately be classified as mild, moderate or severe.

One limitation is the use of the ophthalmologist’s clinical judgment as the gold standard for classifying inflammatory uveitis activity in mild, moderate and severe cases of the disease. Our decision was based on the fact that no gold standard set of criteria for ocular inflammatory activity with which to compare our index currently exists in the scientific literature. In fact, there are some articles in the literature that define the outcome as an “improvement” in activity according to clinical criteria [35–37]. Furthermore, clinical judgment of the physician was also used in the development, of other composite indices as popular as disease activity score (DAS) for rheumatoid arthritis [11]. Another limitation concerning the development phase of the index is the presence of a possible circularity between the predictor variables and the main variable. We will determine whether this potential circularity affects UVEDAI during an already programed validation phase in which patients will be independently evaluated and in a masked form by two ophthalmologists.

Another limitation that had to be overcome involved measuring the model’s discrimination capacity to verify the accuracy of ordinal logistic regression models against existing methods. It must be borne in mind, however, that ordinal logistic regression works under the assumption of proportional odds across responses; that is, each explanatory variable has the same effect at each cumulative split of the ordinal dependent variable (effect of mild vs. moderate + severe ≈ effect of mild + moderate vs. severe). Keeping this in mind, and taking into account that our data did satisfy the proportional odds assumption, the use of two AUCs to measure the capacity to differentiate between patients with mild from those with moderate or severe uveitis, as well as between patients with mild or moderate from those with severe uveitis, gives a good approximation of the total model accuracy.

In conclusion, the UVEDAI is the first ocular easy-to-use composite score based on variables commonly used in clinical practice for uveitis assessment that assesses and classifies global ocular inflammatory activity with high discriminatory power. We believe that it could be a useful tool not only in daily clinical practice, but also for comparing results in clinical and therapeutic studies. Further studies are required to validate this index and analyze its metric capacity.

## Electronic supplementary material

Below is the link to the electronic supplementary material.

Supplementary material 1 (XLSX 11 kb)

Supplementary material 2 (DOCX 15 kb)
