# Supplementary material for: Development of an activity disease score in patients with uveitis (UVEDAI)
Source: Rheumatol Int. 2016 Nov 4;37(4):647–56. doi: 10.1007/s00296-016-3593-1 (PMC5357298; doi:10.1007/s00296-016-3593-1)
Supplement: Supplementary file 2 — Supplementary material 2 (DOCX 15 kb) [file 296_2016_3593_MOESM2_ESM.docx]

**Supplementary Material I**

**Example of a score calculation and associated probabilities**

Example: we want to determine the outcome for a patient who presents a 3+ anterior chamber cell grade, mild vitreous haze, a thickness of 400 μm, no inflammatory vessel sheathing, a patient evaluation of 5, papillitis and 6 chorio-retinal lesions (**Figure 2**):

*Score =1.09 (if anterior chamber cell grade=3) + 0.38 (if vitreous haze=mild) + 1.28 (if macular edema>315) + 0 (if inflammatory vessel sheathing=No) + 0.21*Patient’s evaluation + 1.40 (if papillitis=Yes) + 1.61 (if number of choroidal or retinal lesions≥6)*

*Score= 1.09+0.38+1.28+0+0.21*5+1.40+1.61=6.81*

Therefore, we can calculate the cumulative probabilities:

*Pr (uveitis <Moderate)= 1/(1+e^(6.81-1.01)^)= 0.003*

*Pr (uveitis <Severe)= 1/(1+e^(6.81-4.91)^)= 0.130*

This would facilitate calculation of the patient’s probability to be in each of the categories:

*Pr (uveitis = Mild)=0.003*

*Pr (uveitis = Moderate)= 0.130-0.003=0.127*

*Pr (uveitis = Severe)=1- 0.130=0.870*

This patient would be classified as having severe uveitis.
